# The Status and Prescription Patterns of Opioid Utilization in a Large Comprehensive Teaching Hospital in China According to the Anatomical Therapeutic Chemical Classification/Defined Daily Dose Methodology

**DOI:** 10.3389/fpsyt.2022.913640

**Published:** 2022-05-27

**Authors:** Ting Fang, Xiaojie Zhang, Wei Hao, Qijian Deng

**Affiliations:** ^1^National Clinical Research Center for Mental Disorders, and Department of Psychiatry, The Second Xiangya Hospital, Central South University, Changsha, China; ^2^State Key Laboratory of Toxicology and Medical Countermeasures, Beijing Key Laboratory of Neuropsychopharmacology, Beijing Institute of Pharmacology and Toxicology, Beijing, China

**Keywords:** opioid consumption, prescription pattern, pain management, cancer patient, opioid accessibility, addiction

## Abstract

**Background:**

Few studies have analyzed opioid consumption and the average daily dose and duration for different patients in hospital settings in China. The aim of this study was to measure the status of and trends in prescribed opioids and the prescribing patterns at the Second Xiangya Hospital.

**Methods:**

The data were obtained from the prescribed medicine database of the inpatient department. We included patients who were >18 years old and received any level of opioid analgesic between 2012 and 2017. The international Anatomical Therapeutic Chemical Classification/Defined Daily Dose (ATC/DDD) methodology was used to standardize the consumption rates. All opioid units were converted into morphine equivalents (MEs) to analyze the specific opioid usage.

**Results:**

The consumption of prescribed opioids increased from 3.16 to 3.74 DDD/100 bed-days (+18.3%) from 2012 to 2017. Both cancer and noncancer patients had similar administration routes and median daily dosages in MEs, but cancer patients had longer treatment durations (median: 5 days vs. 1 day, respectively). The median average daily dose and treatment duration for all patients were 15 MEs/day and 2 days, respectively, for oral administration, 100 MEs/day and 1 day for parenteral administration, and 47.14 MEs/day and 5 days for both oral and parenteral administration.

**Conclusion:**

Although there was a tendency toward an increase in opioid consumption, the overall level of consumption in the Second Xiangya Hospital remained relatively low. Thus, it is urgently necessary to increase the availability of opioids and alter prescription habits for them in order adopt the current concept of pain management developed by the World Health Organization (WHO).

## Introduction

The solemn commitment made at the 1961 Single Convention on Narcotic Drugs was amended by the 1972 Protocol and is stated as follows: “To make adequate provisions to ensure and not unduly restrict the availability of drugs that are considered indispensable for medical and scientific purposes.” However, this commitment to opioids has yet to be fulfilled. The most recent data show that in low- and middle-income countries, the incidence of many diseases that require analgesics, especially cancer, is widespread and increasing (1). Opioids are essential for the control of moderate to severe pain, as they can bind to and directly excite opioid receptors in the central nervous system after entering the human body, thereby enhancing or replacing endogenous opioid peptides to regulate pain (2). In the three-step analgesic ladder recommended by the World Health Organization (WHO) for pain relief, patients with moderate to severe pain benefit from the use of opioids (3). However, approximately 5.5 billion people still have limited or no access to narcotic medicines, such as opioid analgesics, meaning that 75% of the world’s population does not have access to proper pain relief treatment (4). In middle- and low-income countries, the prevalence of chronic pain in adults is 33 and 56% in elderly individuals (1). Pain may result in substantial financial burden and can negatively impact quality of life (5).

Opioid use is a two-sided coin that can lead to the development of iatrogenic addiction while treating pain. Unfortunately, global opioid use is unevenly distributed, and approximately 92% of the morphine used worldwide is administered in developed countries, such as the United States, which comprise only 17% of the world’s population (4). According to International Narcotics Control Board’s (INCB’s) report in 2019, developed countries such as the United States have an opioid supply that is large enough to meet more than 1,000% of their demand. Opioid abuse and addiction caused by medical reasons, such as treating chronic pain, have had serious consequences that cannot be underestimated in many countries and regions, especially in the United States. Over the past 15 years, the rate of opioid analgesic use in the United States has soared. From 1999 to 2011, oxycodone consumption increased by almost 500% in the United States. In addition, the mortality rate due to opioid overdose nearly quadrupled. According to the U.S. Centers for Disease Control and Prevention, the unprecedented increase in opioid consumption has led to “the worst drug overdose crisis in U.S. history.” In a speech at the White House on 26 October 2017, U.S. President Donald Trump highlighted the epidemic of opioid abuse in the United States, claiming that opioids kill hundreds of people every day and declaring a national public health emergency. Contrary to the epidemic of abuse in the United States, opioid use for medical purposes in China is extremely inadequate. This unequal distribution of supplies is unequitable to those living in developing countries, as it deprives them of access to medical care, including palliative care. Accordingly, public attention should be given to the imbalance in the availability of opioid analgesics (6).

The medicinal use of opioids in China is quite conservative. China accounts for approximately 20% of the world’s population. In 2016, the consumption of medical morphine in China equaled only 1.8 tons, accounting for 4.98% of global consumption (36.2 tons) (4). According to the 2016 INCB, China’s defined daily doses for statistical purposes (S-DDDs) rank 89th in the world and 22nd in Asia.

According to the WHO’s recommendations, levels less than 200 S-DDDs (per million people per day) are considered inadequate, and levels less than 100 S-DDDs are considered very inadequate. Recent research showed that the total consumption of prescribed opioids in China in 2016 was 78.64 S-DDDs (7). As an essential medication for the control of moderate to severe pain, opioid use is considered an indicator for pain management by the WHO. Thus, pain relief in China is still at a lower level than in other countries.

In China, the number of new cancer patients with severe pathological pain has substantially increased (8). In addition to increasing cancer-related needs, the adequacy of opioid analgesic consumption for severe pain has been lower than the adequacy of consumption measure (ACM) value calculated based on INCB statistics, which ranked it at a “very poor” level from 2006 to 2016. Moreover, a survey conducted in 30 hospitals in Beijing reported that only 9.48% of 589 cancer patients achieved pain relief. To date, there are few convincing clinical data focusing on current opioid-based pain control strategies for patients, and the therapeutic strategy of opioids prescribed for cancer-related pain has not been appropriately addressed in China. Based on these statistics, opioid consumption and the prescription patterns of opioid usage for patients should be analyzed.

Some Chinese studies have focused on the consumption of opioids in hospitals without considering the hospital occupancy index and number of beds. This makes comparisons among different hospitals very difficult. At this critical juncture, China needs not only national data on opioid consumption in terms of S-DDD but also data from hospitals to show the status and trends in opioid prescriptions and related prescribing habits. Collection and analysis of these data are critical for understanding the reasons for low consumption of opioids in China. Hence, the aim of this study was to show the opioid consumption status, trends and prescribing patterns in a teaching hospital as a representative example in China.

## Materials and Methods

### Data Sources

This was a retrospective, descriptive and analytical cross-sectional study performed at the Second Xiangya Hospital, Central South University, in Hunan, China. The data were obtained from the prescribed medicine database of the inpatient department. We extracted the required data from medical records in the hospital database. The collected data included age, sex, hospital ward, diagnosis [the cause of hospitalization, using the 10th revision of the International Classification of Diseases (ICD-10) to determine diagnoses], surgical condition and information about opioid administration, such as the generic name of the opioid analgesic, dosage, route of administration, and treatment duration.

Patients with or without a cancer diagnosis who were aged >18 years and received any opioid analgesic in the 6-year period from 2012 to 2017 were included. Opioid analgesics included parenteral and oral opioids. The parenteral opioids included fentanyl, remifentanil, morphine, tramadol, pethidine, and transdermal fentanyl, and the oral opioids included tramadol (sustained release), oxycodone (sustained release), morphine (sustained release), codeine phosphate, and a combination of paracetamol and tramadol hydrochloride tablets. The study followed the tenets of the Declaration of Helsinki for research involving human subjects, and all participants signed an informed consent form.

### Statistical Analysis

To determine the opioid consumption of the hospital population, the current study used the international Anatomical Therapeutic Chemical Classification/Defined Daily Dose (ATC/DDD) method. The DDD is the assumed average maintenance dose per day for a drug administered for its main indication in adults and is a unit of measurement defined by the WHO Collaborating Centre (WHOCC) for Drug Statistics Methodology (9). DDDs provide a fixed unit of measurement independent of price, currency type, package size, and strength, enabling researchers to assess trends in drug consumption and perform comparisons between population groups. In our study, the DDDs represent the values per 100 inpatients per day and were calculated with the following formula (10):


$$f\left(x\right)=a_{0}+\frac{\begin{array}{c} Number\,of\,units\,delivered\\ in\,afixed\,period\,(mg)\,\times \,100beds \end{array}}{\begin{array}{c} DDD\,(mg)\,\times \,Number\,of\,days\,in\,the\,period\\ \times \,Number\,of\,beds\,\times \,Hospital\,occupancy\,index \end{array}}\,=\,DDD\,/100\,bed\,-\,days$$

To further estimate doctors’ prescribing patterns for different patients, this study converted all oral and parenteral opioid units into morphine equivalents (MEs) for analysis and comparison. The prescribing patterns, including the average daily dose (MEs/day) and the treatment duration, are described with violin plots. Multiple prescriptions for one patient during the 6-year period were considered a complete data record. All statistical analyses were performed using SPSS v. 21.0 for Mac (Chicago, IL, United States), and violin plots were generated with the R (v.3.2.5) program. Values were determined to be significant at *P < 0.05, ^**^P < 0.01, and ^***^P < 0.001.

## Results

A total of 147,814 patients, including 67,343 cancer patients (average of 2.57 prescriptions per patient) and 80,471 noncancer patients (average of 2.50 prescriptions per patient), received opioid treatment during the evaluated time span, based on 374,164 medical records that were extracted.

### The Tendency of Opioid Consumption

**FIGURE 1:**
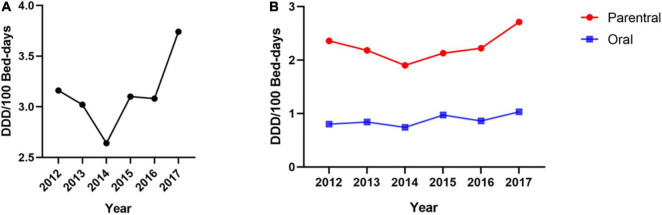
Opioid consumption from 2012–2017 [unit: defined daily dose (DDD)/100 bed-days]. **(A)** The trend of opioid consumption during the 6-year study period. **(B)** The trends of oral and parenteral opioid consumption during the 6-year study period.

As demonstrated in Figure 1A, there was an increasing trend with fluctuations in the consumption of opioids in this hospital. From 2012 to 2014, opioid consumption decreased from 3.16 DDD/100 bed-days to 2.64 DDD/100 bed-days. Then, consumption increased remarkably starting in 2014 and reached 3.74 DDD/100 bed-days in 2017. Figure 1B shows the consumption of oral and parenteral opioids based on DDD/100 bed-day units during the observed 6-year period. From 2012 to 2017, the consumption of parenteral opioids increased by 0.35 DDD/100 bed-days (+14%). For oral opioids, the rate of consumption increased from 0.80 to 1.03 DDD/100 bed-days (+29%).

**FIGURE 2:**
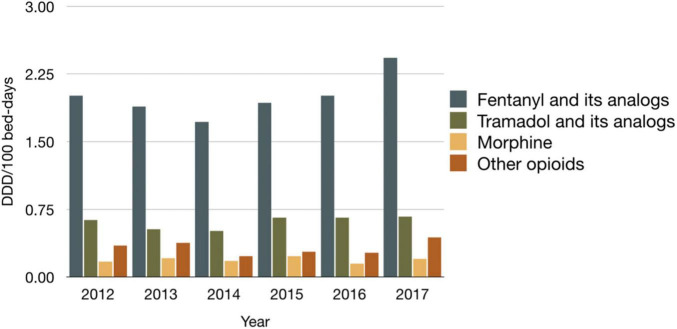
The consumption of all opioids during the 6-year study period.

The top three most consumed opioids were remifentanil, fentanyl, and tramadol, of which remifentanil and fentanyl were administered via the parenteral route and tramadol was administered orally. Figure 2 shows that the consumption of fentanyl and its analogs accounted for a significant proportion (approximately 65%) of the hospital’s total opioid consumption, and remifentanil accounted for 65–86% of the consumption of fentanyl and its analogs from 2012 to 2017.

### The Prescription Patterns of Opioids for Both Cancer and Noncancer Patients

**FIGURE 3:**
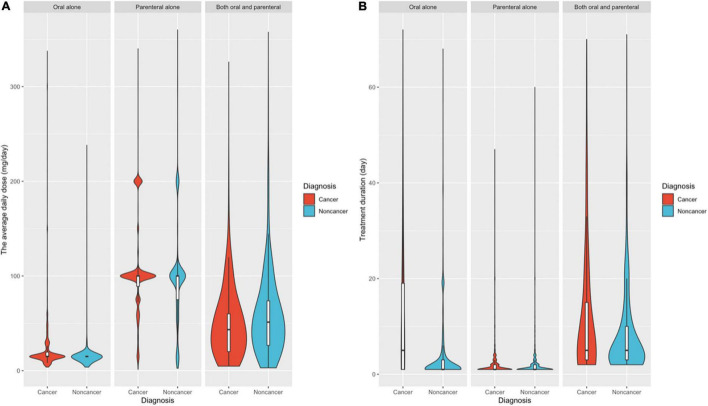
Distribution frequency of the average daily dose **(A)** and treatment duration **(B)** in patients with cancer and noncancer diagnoses.

We classified patients based on their diagnosis as either cancer or noncancer patients, and we used violin plots to evaluate the distributions of daily dosages, treatment durations, etc. Figure 3 shows that both cancer and noncancer patients had similar routes of administration and approximately the same median average daily dose. The following data represent the median dosage and median duration. Among the cancer patients, 5.86% received oral administration (15 MEs/day, 5 days), 87.07% received parenteral administration (100 MEs/day, 1 day), and 7.07% received both oral and parenteral administration (43.33 MEs/day, 5 days). Among the noncancer patients, 6.76% received oral administration (15 MEs/day, 1 day), 79.82% received parenteral administration (100 MEs/day, 1 day), and 13.42% received both oral and parenteral administration (51.25 MEs/day, 5 days).

For all patients with cancer or noncancer diagnoses, the average daily dose for oral administration (median of 15 MEs/day) was significantly lower than that for parenteral administration (median of 100 MEs/day). The average daily dose for patients who used both oral and parenteral opioids (median of 47 MEs/day) was between these two values.

For treatment duration (Figure 3B), the number of days in which a patient received opioids over the 6-year study period and the distribution frequency of different routes of administration were similar. During the 6-year observation period, the treatment duration of noncancer patients (median of 1 day) was significantly lower than that of cancer patients (median of 5 days). Moreover, there was an apparent difference between the treatment duration in the patients receiving only parenteral opioids (median duration of 1 day) and those receiving both oral and parenteral opioids (median duration of 5 days).

The Mann–Whitney test was used to evaluate the differences between the cancer and noncancer groups, including the route of administration, average daily dose and treatment duration. The results showed that all differences between the two groups were significant (*P* < 0.001). However, large sample sizes can easily make small differences in data statistically significant. For this analysis, the differences had limited clinical significance.

## Discussion

There is concern about opioid prescriptions and their potential for global harm, such as addiction (11); however, opioid analgesics are still one of the most effective painkillers. In developing countries, the prevalence of chronic pain in adults is 33%, and 56% in the elderly (1). The effective treatment of people with chronic or cancer-related pain is still limited by an inadequate understanding of the importance of pain therapy or inadequate access to treatment with narcotics (4).

In this large-sample retrospective study, a slight overall increase in opioid consumption was observed in this hospital from 2012 to 2017. However, the consumption of opioids in this hospital did not exceed 4.0 DDD/100 bed-days during the whole 6-year observation period. Based on the doctors’ prescribing patterns, the average daily dose for both cancer and noncancer patients was low for the different administration routes, and the treatment durations were short, especially for cancer patients, at only 5 days.

Given that there is no international standard for measuring the level of hospital opioid consumption, comparisons were made between our collected data and existing foreign published studies on drug utilization and opioid consumption evaluations in hospital settings based on the ATC/DDD system. We confirmed the hypothesis of our study, that is, opioid consumption at this hospital was relatively low compared with the consumption rates reported in other studies, which included two hospitals and four databases from Europe, East Asia, and North America (12–17). One study using the same measurement unit (DDD/100 bed-days), carried out in a hospital in Madrid, Spain, revealed that opioid consumption had a remarkable increasing tendency, from 22.3 DDD/100 bed-days in 2011 to 26.5 DDD/100 bed-days in 2015 (16). Another study in Iran evaluated parenteral opioid analgesic utilization in a referral teaching hospital in 2013 (17) and the number of patients who received parenteral opioid analgesics (41.185 DDD/100 bed-days) was nearly 21.9 times higher than that in this hospital (1.88 DDD/100 bed-days) in 2013. As a large, comprehensive, university-affiliated hospital, the authors believe that the consumption of opioids in this hospital must be greater than that in other local hospitals. The low level of opioid consumption was also consistent with national data (4), which show that the consumed number of opioids (<100 S-DDDs) is relatively low compared with the rest of the world.

The low level of opioid consumption in China may be attributable to multiple factors, such as policies, regulations, culture, and awareness (18). As the WHO report stated, the Chinese government’s fear of opioid abuse could be one reason for the limited availability of medical opioids (4). Recent research has revealed that there is no evidence showing a precise relationship between high medical opioid consumption and the abuse of prescription opioids (19). Additionally, the fear of opioid abuse is due to an insufficient understanding of opioids and a lack of practical and reasonable clinical guidelines. According to research conducted in China that administered the Knowledge and Attitudes Survey Regarding Pain (KASRP) to doctors and nurses, the doctors and nurses did not receive passing scores (the authors of this questionnaire suggested that a score of 80% was considered a passing score) (20). Finally, China has a unique history. By the beginning of the 20th century, 85–95% of the total global opium was consumed and misused by Chinese people (18). As a result, both patients and their family members fear nonmedical use, addiction to opioids, and opioid-induced side effects, which may have led to insufficient medical use (21).

In a study assessing the basic knowledge of cancer pain management in a Chinese hospital, oncologists correctly answered only 59.7% of the knowledge questions (22), while in various other studies, oncologists scored between 31 and 68% (23–25), indicating an insufficient understanding of opioid use for cancer patients in this hospital. In this study, cancer patients received similar daily doses and had similar routes of administration, which may not be consistent with the clinical treatment guidelines. Both cancer and noncancer patients had similar prescription patterns. Last but not least, the treatment duration for all patients was relatively short, especially for cancer patients. The reason may be related to the fear of side effects and drug addiction. These fears are in accordance with the findings by Jeon et al. (26), who reported that 90.6% of doctors worried about difficulties in controlling the side effects of strong opioids.

Finally, only the sustained-release formulations of oral opioids were administered in this hospital. The WHO guidelines recommend the use of immediate-release opioid formulations in the early stage of treatment to enable rapid titration to the optimal dose for individualized treatment. The lack of an immediate-release formulation means that titration to the optimal dose cannot be accurately achieved.

### Recommendations for Rationalizing Opioid Use in China

The two extremes of the opioid crisis, overuse and the lack of analgesia caused by underuse, demonstrate the difficulty of opioids as a formal medical method of analgesia in clinical application. Whether it is opioid abuse in the United States or China’s lack of analgesia, the essence of the problem is that a balance between medical use and the prevention of abuse has not been found. Based on the analysis of existing research results, the authors believe that the following recommendations should be employed to improve the accessibility and rational administration of opioids:

1. Legislation, monitoring, and advocacy: comprehensive and balanced regulations to address the rational use of opioids and addiction-related problems should be adopted. The medical use of opioids, especially for palliative care for cancer patients, should not be restricted because of opioid addiction concerns. Moreover, based on lessons learned from the opioid crisis in North America (27), controlling the activities of pharmaceutical enterprises and strengthening drug monitoring after marketing are very important.
2. Updated guidance: the current guidance for opioid prescriptions and pain treatment should be updated based on international experiences, scientific evidence, and clinical practice.
3. Professional training: professional training should include comprehensive knowledge of relevant topics, such as pain management, pharmacological effects, and the addiction potential of opioids, as well as the early identification, assessment, and treatment of opioid use-related abuse and addiction.
4. Establishment of a government coordination program: it is important to have a coordinated program for pain management, opioid prescription management and drug abuse prevention to balance the treatment needs of pain patients and opioid addiction and diversion.
5. Public education: public education should include overcoming the fear of opioid use by providing scientific information on the rational use of opioids to change knowledge and attitudes toward opioids and the risk of opioid addiction.

### Limitations

This study has limitations. The findings are based only on medical records used to represent overall opioid use in this hospital. The authors are not aware of individual pain relief responses without further investigation of the patients. Additionally, this study only described part of the current situation in one hospital, and further research is needed to provide a complete picture and guide clinical practice.

## Conclusion

Our study demonstrated that despite a positive trend in 2012–2017, opioid consumption in this hospital was still at a relatively low level. Both cancer and noncancer patients had similar administration routes and dosages, but the cancer patients had a longer treatment duration (median: 5 days vs. 1 day, respectively). The median average daily dose and treatment duration for all patients were 15 MEs/day and 2 days for those who received oral administration, 100 MEs/day and 1 day for those who received parenteral administration, and 47.14 MEs/day and 5 days for those who received both oral and parenteral administration, respectively. The authors also found that the cancer patients did not receive personalized pain management. We should recognize that opioids are indispensable for medical and scientific purposes and that the availability of opioids for such purposes should not be excessively restricted. China’s regulations, the availability of opioids and the management of access to pain relief must be further strengthened. Following this, guidelines for personalized pain treatment should be established for patients with different diagnoses.

## Data Availability Statement

The original contributions presented in the study are included in the article/supplementary material, further inquiries can be directed to the corresponding author.

## Ethics Statement

The studies involving human participants were reviewed and approved by Ethics Committee of the Second Xiangya Hospital, Central South University. Written informed consent for participation was not required for this study in accordance with the national legislation and the institutional requirements.

## Author Contributions

TF and WH designed the study and wrote the manuscript. TF, XZ, and QD performed the experiments and analyzed the data. All authors contributed to the article and approved the submitted version.

## Conflict of Interest

The authors declare that the research was conducted in the absence of any commercial or financial relationships that could be construed as a potential conflict of interest.

## Publisher’s Note

All claims expressed in this article are solely those of the authors and do not necessarily represent those of their affiliated organizations, or those of the publisher, the editors and the reviewers. Any product that may be evaluated in this article, or claim that may be made by its manufacturer, is not guaranteed or endorsed by the publisher.
